# Downregulation of SOX2-OT Prevents Hepatocellular Carcinoma Progression Through miR-143-3p/MSI2

**DOI:** 10.3389/fonc.2021.685912

**Published:** 2021-07-12

**Authors:** Hongfeng Zhao, Minping Bi, Meng Lou, Xiaowei Yang, Liwen Sun

**Affiliations:** Department of Oncology, Xinxiang Central Hospital, The Fourth Clinical of Xinxiang Medical University, Xinxiang, China

**Keywords:** hepatocellular carcinoma, LncRNA SOX2-OT, miR-143-3p, MSI2, CeRNA, proliferation, migration, invasion

## Abstract

**Objective:**

LncRNA SOX2-OT is involved in a variety of cancers. This study explored the effect of lncRNA SOX2-OT on hepatocellular carcinoma (HCC) cells.

**Methods:**

SOX2-OT expressions were detected in HCC tissues and normal tissues, normal cells, and HCC cells. The relationship between SOX2-OT and prognosis was analyzed by TCGA. After SOX2-OT expression was inhibited using siRNA, HCC cell malignant behaviors were evaluated. The subcellular localization of SOX2-OT in HCC cells was predicted and analyzed. The binding relationships among SOX2-OT, miR-143-3p, and MSI2 were analyzed by bioinformatics website, dual-luciferase assay, and RNA pull-down assay. The effect of miR-143-3p and MSI2 on the regulation of SOX2-OT on biological behaviors of HCC cells was confirmed by functional rescue experiments. The effect of SOX2-OT on the tumorigenicity of HCC was evaluated by subcutaneous tumorigenesis in nude mice.

**Results:**

SOX2-OT was highly expressed in HCC cells and tissues. The prognosis was poor in HCC patients with high SOX2-OT expression. Downregulating SOX2-OT inhibited HCC cell malignant behaviors. SOX2-OT bound to miR-143-3p to promote MSI2 expression. Downregulating miR-143-3p or upregulating MSI2 averted the role of si-SOX2-OT in HCC cells. Nude mouse subcutaneous tumorigenesis showed that SOX2-OT downregulation decreased the tumorigenicity of HCC, and affected the levels of miR-143-3p and MSI2 mRNA in tumor tissues.

**Conclusion:**

SOX2-OT inhibited the targeted inhibition of miR-143-3p on MSI2 through competitively binding to miR-143-3p, thus promoting MSI2 expression and proliferation, invasion, and migration of HCC cells.

## Introduction

Primary carcinoma of the liver is one of the most prevalent cancers and also a most common cause of cancer mortality all over the world (1). The incidence rate of liver cancer and liver cancer-related deaths has been increasing for decades (2). Hepatocellular carcinoma (HCC) accounts for the majority of primary liver malignancies (3). The development of HCC is related to chronic viral infection (mainly HBV and HCV). HCC is characterized by multicentric cancerization, intrahepatic metastasis, and multistage cancerization (4). As HCC arises in the body of chronic liver disease, its prognosis is determined by the underlying disease, and the therapeutic options are therefore limited in late stages (5). The possibility of early detection is very small if any. Therefore, the need to clarify the precise mechanisms of HCC development is very strong (6).

Long non-coding RNA (LncRNA) is an RNA transcript that doesn’t encode any protein (7). LncRNAs have an essential effect on gene regulation and intracellular homeostasis, including cell proliferation, survival, and migration (8). Primarily, lncRNAs have the property to influence the metastatic tumor microenvironment and the occurrence and progression of various cancers (9). Thousands of lncRNAs are differentially expressed in HCC, which makes them potential biomarkers and possible targets for HCC management (10, 11). SOX2-OT is upregulated in nasopharyngeal carcinoma and SOX2-OT upregulation is related to the poor outcomes of nasopharyngeal carcinoma (12). The expression of SOX2-OT is significantly elevated in prostate cancer cells and tissues, and lncRNA SOX2-OT facilitates the migration and proliferation of prostate cancer cells (13). LncRNA SOX2-OT facilitates the development of laryngeal squamous carcinoma (14). LncRNA SOX2-OT hinders the therapy and deteriorates the prognosis in malignant lung diseases (15). However, there are limited reports on the lncRNA SOX2-OT in HCC and its mechanism.

Competing endogenous RNAs (ceRNAs) are the transcripts that modulate each other at the post-transcriptional level by competing to share microRNAs (miRs) (16). The previous study has shown that lncRNAs can bind to miRs as ceRNAs and regulate the expression of miRs and target genes (17). CeRNAs participate in various biological processes in HCC cells, such as cell proliferation, invasion, and chemoresistance (18, 19). miRs act as regulators of gene expression through binding to mRNAs directly and cause the inhibition of mRNA translation or the degradation of target genes (20), and multiple miRNAs have been reported to be significantly associated with overall survival in HCC (21). A recent study indicates that SOX2-OT acts as a ceRNA to regulate miR-144-3p expression (22). miR-143-3p may block HCC cell proliferation (23). Moreover, it is found out that HCC cell invasion is significantly decreased by the knockdown of MSI2 (24). The target relationship of MSI2 and miR-143-3p has been verified (25). But the network of SOX2-OT/miR-143-3p/MSI2 in HCC remains unstudied.

At present, whether lncRNA SOX2-OT can modulate MSI2 expression by regulating miR-143-3p and then affect the malignant behaviors of HCC cells haven’t been studied at home and abroad. This study investigated the lncRNA SOX2-OT expression and its mechanism in HCC, to provide new ideas for HCC treatment.

## Materials and Methods

### Ethics Statement

All procedures were authorized and supervised by the academic ethics committee of Affiliated Xinxiang Central Hospital of Xinxiang Medical University. Informed consent was signed by each eligible participant. The animal experiment in this study was approved by the Medical Laboratory Animal Management Committee of Affiliated Xinxiang Central Hospital of Xinxiang Medical University. All procedures were strictly implemented according to Animal management regulations. All the laboratory procedures were used to reduce the pain of the mice, such as heating pads, disinfection, and replenishing fluids with saline.

### HCC Tissue Collection

The postoperative specimens of HCC tissues and normal tissues were selected from 87 cases of patients with HCC who accepted pathological resection in the Department of oncology of Affiliated Xinxiang Central Hospital of Xinxiang Medical University from January 2014 to March 2019. The average age of HCC patients was 53 years (41-72 years). The inclusion criteria were that the clinical data of patients were complete, all patients did not have hepatitis caused by other types of hepatitis virus infection except hepatitis B virus, and did not receive tumor treatments of targeted drug therapy and local ablation therapy before surgery Important clinicopathological parameters of patients were shown in **Table 1**.

**Table 1:** Clinical features of HCC.

Clinical variables	No. or value of specimens (n = 87)
Age (year, mean ± SD)	50.6 ± 11.2
Male (N)	69
Female (N)	18
Child-Pugh class	
A (N)	72
B (N)	15
Cirrhosis (N)	73
AFP, ng/ml	
<20 (N)	31
20-100 (N)	13
100-400 (N)	9
>400 (N)	34
Portal vein tumor thrombus (N)	52
Tumor size, cm	
<3	15
March 5th	29
>5	43
TNM staging	
Stage I (N)	49
Stage II (N)	23
Stage III (N)	15

TNM staging was performed according to the AJCC Cancer Staging Manual, 7th Edition (2010) published by Springer International Publishing.

### Bioinformatics Analysis

The downstream target genes of lncRNA SOX2-OT were predicted using ENCORI (http://starbase.sysu.edu.cn/) (26) and DIANA Tool (https://diana.e-ce.uth.gr/lncbasev3/interactions) (27), and the intersection was obtained. The downstream target genes of miRNA were predicted using the RNAInter (http://www.rna-society.org/rnainter/), Targetscan (http://www.targetscan.org/vert_71/), and miRDB (http://www.mirdb.org/), and the intersection was obtained.

### Cell Culture

Human HCC cell lines HepG2, Hep3B, HCCLM3, SMMC-7721, and Huh7 and human normal hepatocyte line L02 from ATCC cell bank (ATCC, Manassas, VA, USA) were all cultured in DMEM (Gibco, Carlsbad, CA, USA) containing 10% fetal bovine serum, 100 U/mL penicillin and 100 μg/mL streptomycin (Gibco) at 37°C with 5% CO_2_.

### Cell Transfection

HepG2 and Huh7 cells were seeded into 6-well plates (2 × 10^5^/well). After 24 hours of incubation, cells were transfected with si-NC, si-SOX2-OT#1, si-SOX2-OT#2, si-SOX2-OT#3, mimic NC, miR-143-3p mimic, inhibitor NC, miR-143-3p inhibitor, pcDNA3.1-NC, or pcDNA3.1-MSI2 (Genechem, Shanghai, China) (siRNA 10-100 nM, miRNA-inhibitor 50 nM, pcDNA3.1 10 nM) 48 hours using Lipofectamine 2000 (11668-019, Invitrogen, Carlsbad, CA, USA). The siRNA sequences were as follows: si-SOX2-OT#1 (5′-CAAGACAACACCCTGATCT-3′); si-SOX2-OT#2 (5′-GCCAATCAAACTGCTACAA-3′), si-SOX2-OT#3 (5′-GAACTGCAAGCTCCTTCAA-3′), si-negative control (5′- UUCUCCGAACGUGUCACGU-3′). miR-143-3p mimic (5′-UGAGAUGAAGCACUGUAGCUC-3′), miR-143-3p inhibitor (5′-GAGCUACAGUGCUUCAUCUCA-3′); The open reading frame of MSI2 gene was amplified using forward primer: 5′-GATATGGAGGCAAATGGGAGC-3′ and reverse primer: 5′-GTAATTCCCCACTCCGGAGTCCTG-3′. The PCR product was purified and directly cloned into the pcDNA3.1 vector.

### Reverse Transcription Quantitative Polymerase Chain Reaction (RT-qPCR)

TRIzol reagent (Invitrogen) was used to extract the total RNA from clinical tissues or cultured cells following the instructions. PrimerScript RT Master Mix (Takara, Dalian, China) was applied for reverse transcription of 1 ug RNA into cDNA. Subsequently, SYBR Premix Ex Taq (Takara) was used for RT-qPCR on ABI 7500 (ABI, Foster City, CA, USA). GAPDH and U6 were internal parameters and the 2^-△△Ct^ method was used to calculate the relative expression of genes. PCR primers are shown in **Table 2**.

**Table 2:** Primer sequence of RT-qPCR.

Name of primer	Sequences
*SOX2-OT*-F	GTTCATGGCCTGGACTCTC C
*SOX2-OT*-R	ATTGCTAGCCCTCACACCTC
U6-F	GCTTCGGCAGCACATATACTAAAAT
U6-R	CGCTTCACGAATTTGCGTGTCAT
*miR-143-3p*-F	CTGGCGTTGAGATGAAGCAC
*miR-143-3p* -R	CAGAGCAGGGTCCGAGGTA
*miR-369-3p*-F	TGACCTAAGGGACTCCCACAA
*miR-369-3p* -R	TAGCAATATTGCACAGAAGGC
GAPDH-F	TGCACCACCAACTGCTTAGC
GAPDH-R	GGCATGCACTGTGGTCATGAG
MSI2-F	ACCTCACCAGATAGCCTTAGAG
MSI2-R	AGCGTTTCGTAGTGGGATCTC

### Western Blot

The cells were added with radioimmunoprecipitation assay (RIPA) lysate (Beyotime, Shanghai, China) containing protease inhibitor cocktail (Sigma-Aldrich, St. Louis, MO, USA), mixed well and lysed on ice for 30 min. After the cells were centrifuged for 10 min, the supernatant was collected. The protein concentration was detected using the bicinchoninic acid (BCA) kit. The diluted samples were added with loading buffer, boiled at 100°C, and denatured. The protein was separated using 100 V constant sodium dodecyl sulfate-pressure polyacrylamide gel electrophoresis (SDS-PAGE) and transferred to nitrocellulose membranes at 4°C. Next, the membranes were added with 70 g/L skim milk powder, incubated on a shaker at room temperature, and sealed for 1 h. The membranes were washed on a shaker using tris buffered saline and Tween-20 (TBST) for 5 min 3 times. The membranes were incubated with primary antibody rabbit anti-MSI2 (1:1000, ab76148, Abcam, Cambridge, MA, USA) at 4°C overnight, with secondary antibody (1:2000, ab205718, Abcam) at room temperature for 2 h, and washed on a shaker using TBST for 5 min for 3 times. Then, the membranes were added with enhanced chemiluminescence (ECL) for development and scanned in a dark room using a scanner. β-actin (1:1000, ab252556, Abcam) was used as the loading control. The grayscale of electrophoresis bands was analyzed using ImageJ software.

### Cell Counting Kit-8 (CCK-8)

The cells were plated in 96-well plates (3000 cells/well) and cultured at 37°C. After incubation for 0, 24, 48, and 72 hours, 10 μL CCK-8 reaction reagent (Beyotime) was added to each well. After incubation at 37°C for 2 hours, the absorbance was detected at a wavelength of 450 nm and the cell proliferation curve was drawn.

### Colony Formation Assay

The cells were detached using trypsin, collected and seeded into 6-well plates at 10^3^ cells/well, and incubated in RPMI-1640 medium at 37°C for 2 weeks. The medium was refreshed every 3 days. All cells were treated with 4% paraformaldehyde and stained with gentian violet solution. The cell colonies with more than 50 cells were counted and photographed using a digital camera (Olympus, Tokyo, Japan).

### Transwell Assay

After transfection for 48 h, HepG2 and Huh7 cells were collected. The cell density was adjusted to 1 × 10^6^/mL after resuspension. In the invasion assay, 50 mg/L Matrigel was diluted at a ratio of 1:8 and then spread on the chamber bottom; no Matrigel was applied in the migration assay. A total of 600 μL complete medium was added into the basolateral chamber, and 200 μL cell suspension was paved in the apical chamber. Three samples were repeated in each group. After 24 h, the chamber was removed and cleared with the phosphate buffer saline (PBS) 3 times, and the Matrigel and cells on the upper layer of the microporous membrane of the chamber were wiped gently using the cotton swab. The chamber was then fixed with paraformaldehyde (40 g/L, 15 min) and stained with crystal violet (1 g/L, 10 min). At last, the cells were observed and counted using the inverted microscope. Six visual fields were randomly selected from each sample to evaluate the invasion and migration ability of cells.

### Subcutaneous Tumorigenesis in Nude Mice

The 3-4-week old BALB/C nude mice, weighing 15-18 g, were purchased from Beijing Vital River Laboratory Animal Technology Co., Ltd., [(SYXK) (Beijing) 2016-0011]. In the experiment, nude mice were raised at 26-28°C with a relative humidity of 40-60% in a specific pathogen-free (SPF) environment in different cages for 10/14-hour light/dark cycles with freely available food and water. The nude mice were fed adaptively for 1 week before the experiment. HepG2 cells stably interfering with lncRNA SOX2-OT and the control cells were detached using trypsin and counted. Totally 5 ×10^6^ cells were suspended in 100 μL PBS. The cell suspension was subcutaneously injected into the lateral abdomen of nude mice, with 6 nude mice in each group. The growth of the tumor was observed every 4 d, and the volume of the tumor was measured using a vernier caliper. The nude mice were killed 4 weeks after the injection of cancer cells. The tumor was collected and weighed for the following experiments.

### Nuclear/Cytosol Fractionation Assay

The cell nucleus and cytoplasm were separated using Nuclear and cytoplasmic protein extraction kits (Beyotime). The cells were washed with PBS buffer and then incubated in 200 μL cytoplasmic protein extractor A/protease inhibitor buffer, and placed on ice for 10-15 min. The cells were then incubated in 10 μL cytoplasmic protein extract B and centrifuged at 12000 g at 4°C for 10 min so that the nuclei precipitation was far away from the cytoplasm. The nucleus was resuspended in 50 μL nucleoprotein extraction buffer and stirred on ice for 30 min. After centrifugation at 12000 g at 4°C for 10 min, the supernatant was collected as the nuclear extract for subsequent RT-qPCR analysis.

### RNA Fluorescence *In Situ* Hybridization (FISH) Assay

HepG2 and Huh7 cells were fixed with 4% formaldehyde for 15 min and then washed with PBS buffer. The fixed cells were treated with pepsin (1% 10 mmol/L HCl) and then dehydrated by 70%, 90%, and 100% ethanol. The cells after air-dry were cultured with 40 nmol/L SOX2-OT probes in hybridization buffer (100 mg/mL dextran sulfate, 2×10% formamide in SSC) at 80°C for 2 min, and hybridized at 55°C for 2 h. The slides were washed and dehydrated and stained with 1 mg/mL DAPI for 10 min after air-dry. RNA FISH probe was designed and synthesized by Bogu Co, Ltd (Shanghai, China) (5’FITC-TCATACTTTGAAGGGATTGCAGTGG-3’). The results were analyzed by Nikon inverted fluorescence microscope.

### Dual-Luciferase Reporter Assay

Starbase was applied to predict and analyze the binding sites of SOX2-OT and miR-143-3p, and Targetscan was used to predict and analyze the binding sites of miR-143-3p and MSI2. The wild-type (SOX2-OT-wt and MSI2-wt) and mutant-type (SOX2-OT-mut and MSI2-mut) luciferase plasmids were constructed by cloning the binding and mutated sequences into the luciferase vector pGL3 (Promega, Madison, WI, USA). The constructed plasmids were cotransfected with miR-143-3p mimic or mimic NC into HepG2 and Huh7 cells. The luciferase activity was evaluated using the Dual-Lucy assay kit (Solarbio, Beijing, China) after 24 h.

### RNA Pull-Down

SOX2-OT and the standard RNA were labeled by 3’ End Biotinylation kit (Thermo, Waltham, MA, USA). Biotin-labeled RNA pull-down assay was performed for the detection of miRs interacting with each other. The biotin-labeled RNA and streptavidin magnetic beads were placed in the capture buffer and stirred for 30 min. The lysate was collected and mixed with magnetic beads combined with SOX2-OT in a 4°C rotator for 4 h. After sufficient washing of the magnetic beads, the RNA bound to the magnetic beads was isolated and analyzed by RT-qPCR. The probe sequences were as follows: Biotin-NC (5’-Biotin-UUCUCCGAACGUGUCACGUTT-3’); Biotin-SOX2-OT-wt (5’-Biotin-UCGCCUGGCAAGAUCAUCUCA-3’); Biotin-SOX2-OT-mut (5’-Biotin-UCGCCUGGCAAGAAGUAGAGA-3’).

### Statistical Analysis

SPSS21.0 software (IBM Corp. Armonk, NY, USA) and GraphPad Prism 8.0 software (GraphPad Software Inc, San Diego, CA, USA) were used for data analysis and mapping. All data were shown as mean ± standard deviation. *T*-test was used for comparisons between two groups, and one-way analysis of variance (ANOVA) was used for comparisons of multi-groups, followed by Tukey’s multiple comparisons test. The *P*-value was obtained by a bilateral test. A value of *p* < 0.05 indicated statistical significance.

## Results

### SOX2-OT Was Upregulated in HCC

**Figure 1:**
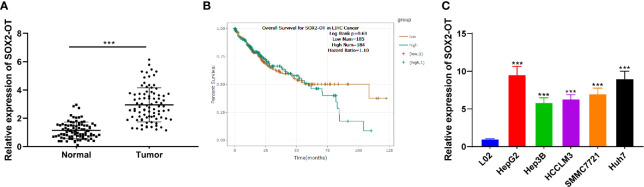
SOX2-OT was elevated in HCC. **(A)** SOX2-OT expression in HCC tissues and normal tissues of HCC patients was detected by RT-qPCR, N = 87; **(B)** The relationship of SOX2-OT expression and prognosis was analyzed through TCGA visualization Starbase website (http:starbase.sysu.edu.cn); **(C)** SOX2-OT expression in HCC cells and normal hepatocytes was examined by RT-qPCR. The cell experiment was repeated three times independently, and the data were all metrological data. All data were shown as mean ± standard deviation. T test or one-way ANOVA was used for data analysis, followed by Tukey’s test. ***P < 0.001.

To explore the role of SOX2-OT in HCC, RT-qPCR was used to analyze the SOX2-OT expression in HCC tissues and normal tissues of patients with HCC. SOX2-OT in HCC tissues was much higher than that in paracancerous tissues (*p* < 0.001, **Figure 1A**). In addition, the TCGA visualization website Starbase (http:starbase.sysu.edu.cn) was used to analyze the relationship between SOX2-OT expression and prognosis. The results showed that although there was no significant difference in the K-M curve of patients with different SOX2-OT expressions, the long-term prognosis of patients with low SOX2-OT expression showed a better trend (**Figure 1B**). Subsequently, HCC cell lines HepG2, Hep3B, HCCLM3, SMMC7721 and Huh7, and human normal hepatocyte line L02 were selected, and the expression difference of SOX2-OT was detected by RT-qPCR. SOX2-OT in HCC cell lines was much higher than that in normal human hepatocyte line L02 (all *p* < 0.001, **Figure 1C**). The results above suggested that SOX2-OT might be related to HCC.

### SOX2-OT Downregulation Prevented Invasion and Proliferation of HCC Cells

**Figure 2:**
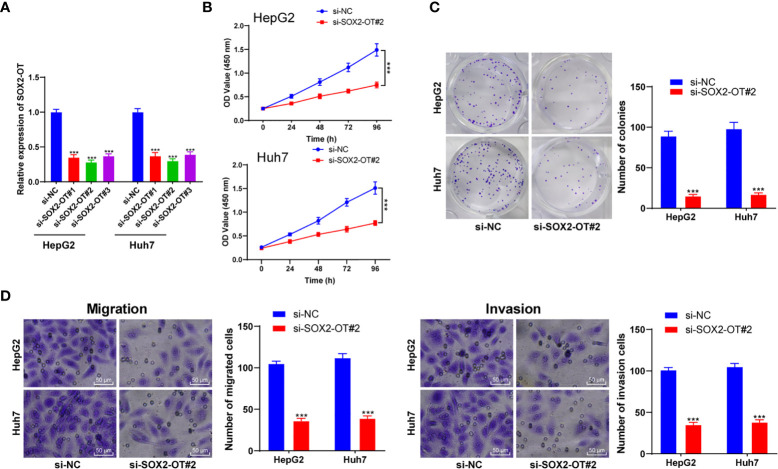
Downregulation of SOX2-OT inhibited proliferation and invasion of HCC cells. SOX2-OT was intervened by siRNA (si-SOX2-OT#1, si-SOX2-OT#2, si-SOX2-OT#3) in HepG2 and Huh7 cells. The transfection of si-NC was used as control, and **(A)** the intervention effect of si-SOX2-OT was verified by RT-qPCR; the effects of si-SOX#2-OT2 with the best interference efficiency on cell proliferation, migration, and invasion were detected by **(B)** CCK-8 and **(C)** colony formation assay and **(D)** Transwell assays. Cell experiment was repeated three times independently and data were all metrological data. All data were shown as mean ± standard deviation. One-way ANOVA was used for data analysis, followed by Tukey’s test. ***P < 0.001.

To identify the role of SOX2-OT in HCC cells, HepG2 and Huh7 cells with relatively high expression were selected according to the results in **Figure 1C**, and the SOX2-OT expression was inhibited by transfection of SOX2-OT siRNA, and the si-NC transfection was used as the control. Firstly, RT-qPCR demonstrated that SOX2-OT expression was decreased clearly after the transfection of SOX2-OT siRNA (all *p* < 0.001, **Figure 2A**). Si-SOX2-OT#2 with the best inhibition efficiency was selected for the following experiments. Subsequently, CCK-8 and colony formation assays demonstrated that downregulation of SOX2-OT significantly prevented the proliferation of HepG2 and Huh7 cells (all *p* < 0.001, **Figures 2B, C**). Transwell assays indicated that the migration and invasion were also inhibited after downregulation of SOX2-OT (all *p* < 0.01, **Figure 2D**). Briefly, downregulation of SOX2-OT could inhibit malignant behaviors of HCC cells.

### SOX2-OT Regulated MSI2 Expression by Combing With miR-143-3p

**Figure 3:**
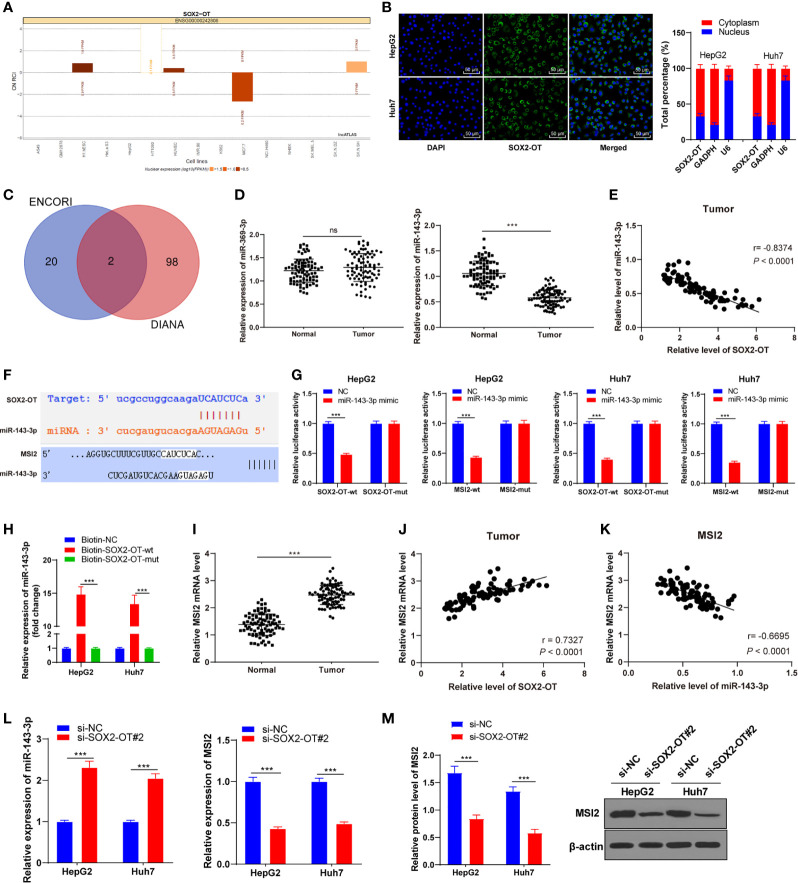
SOX2-OT regulated MSI2 expression by binding to miR-143-3p. **(A)** A bioinformatics website (lncatlas.crg.eu) was used to predict, and the nuclear/cytosol fractionation assay was used to verify the subcellular localization of SOX2-OT; **(B)** The distribution of SOX2-OT in nucleus and cytoplasm was detected by the RNA-FISH assay; **(C)** The downstream target genes of lncRNA SOX2-OT were predicted using ENCORI and DIANA Tool, and the intersection was obtained; **(D)** miR-369-3p and miR-143-3p levels in cancer tissues and adjacent normal tissues of HCC patients were detected by RT-qPCR, N = 87; **(E)** The correlation between SOX2-OT and miR-143-3p in cancer tissues and adjacent normal tissues of HCC patients was analyzed using Pearson coefficient; **(F)** The binding sites of SOX2-OT and miR-143-3p, and miR-143-3p and MIS2 were predicted using Targetscan and Starbase; **(G)** The target relationship of SOX2-OT and miR-143-3p, and miR-143-3p and MIS2 were verified by dual-luciferase assay; **(H)** The target relationship of SOX2-OT and miR-143-3p was detected by RNA Pull-down assay; **(I)** The mRNA level of MSI2 in cancer tissues and adjacent normal tissues of HCC patients was detected by RT-qPCR; **(J)** The correlation between SOX2-OT and MSI2 mRNA in cancer tissues of HCC patients was analyzed using Pearson coefficient; **(K)** The correlation between miR-143-3p and MSI2 mRNA in cancer tissues of HCC patients was analyzed using Pearson coefficient; **(L)** The expressions of miR-143-3p and MSI2 in HepG2 and Huh7 cells with si-SOX2-OT#2 were detected by RT-qPCR; **(M)** The protein level of MSI2 in HepG2 and Huh7 cells transfected with si-SOX2-OT#2 was determined by western blot. The cell experiment was repeated three times independently and data were all metrological data. All data were shown as mean ± standard deviation. One-way ANOVA was used for data analysis, followed by Tukey’s test. ***P < 0.001, ns (no statistical significance).

To further understand the potential mechanism of SOX2-OT in HCC, a bioinformatics website (lncatlas.crg.eu), and the nuclear/cytosol fractionation assay and RNA-FISH assay were used to verify the subcellular localization of SOX2-OT in HCC cells. It was found that SOX2-OT was mainly distributed in the cytoplasm of HepG2 and Huh7 cells (all *p* < 0.001, **Figures 3A, B**). According to this, we speculated that SOX2-OT might act as a ceRNA in the biological processes of HCC cells. To further study the ceRNA mechanism of SOX2-OT, the downstream target genes of lncRNA SOX2-OT were predicted using ENCORI and DIANA Tool, and the intersections hsa-miR-369-3p and hsa-miR-143-3p were obtained after screening (**Figure 3C**). miR-143-3p has been reported to be involved in the biological behaviors of HCC (28). The levels of two miRNAs in HCC tissues and adjacent normal tissues were analyzed by RT-qPCR and the results showed that there was a significant difference in miR-143-3p expression (*P* < 0.001, **Figure 3D**), and miR-143-3p was negatively correlated with lncRNA SOX2-OT (*P* < 0.001, **Figure 3E**). Therefore, miR-143-3p was selected as the downstream target of SOX2-OT. Starbase and Targetscan databases predicted that there were binding sites between SOX2-OT and miR-143-3p, and between miR-143-3p and MSI2 (**Figure 3F**). MSI2 may be a potential molecular target for the treatment of HCC (29). Therefore, we speculated that miR-143-3p/MSI2 might regulate the behavior alternation of HCC cells. Subsequently, the binding relationship of miR-143-3p and MSI2 was verified by dual-luciferase assay and RNA pull-down assay (all *P* < 0.001, **Figures 3G, H**). The mRNA level of MSI2 was significantly upregulated in HCC tissue compared to adjacent normal tissues in HCC patients (*P* < 0.001, **Figure 3I**), and was positively correlated with lncRNA SOX2-OT (*P* < 0.001, **Figure 3J**), and negatively correlated with miR-143-3p (*P* < 0.001, **Figure 3K**). RT-qPCR demonstrated that compared with the si-NC group, the expression of miR-143-3p in HepG2 and Huh7 cells transfected with si-SOX2-OT#2 was significantly upregulated, while the expression of MSI2 was significantly downregulated (all *P* < 0.001, **Figure 3L**). The protein level of MSI2 was detected by western blot. It was found that the protein level of MSI2 in HepG2 and Huh7 cells transfected with si-SOX2-OT#2 was decreased (*P* < 0.001, **Figure 3M**), indicating that SOX2-OT competitively bound to miR-143-3p and promoted MSI2 expression.

### Downregulation of miR-143-3p Averted the Effect of si-SOX2-OT on HCC

**Figure 4:**
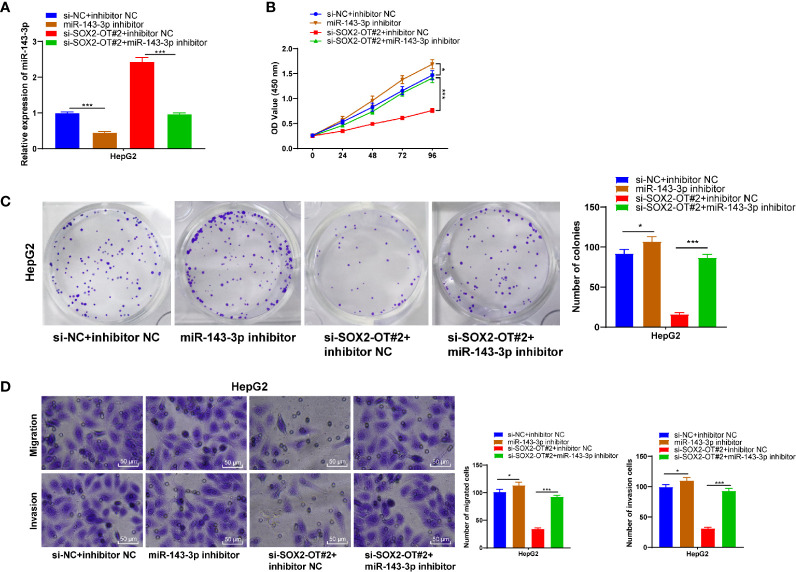
Downregulation of miR-143-3p could reverse the inhibitory effect of si-SOX2-OT on HCC. The miR-143-3p inhibitor was delivered into HepG2 cells with si-SOX2-OT#2, and the cells with si-NC+inhibitor-NC were used as controls. Then, **(A)** RT-qPCR was used to verify the intervention effect of miR-143-3p inhibitor; **(B)** CCK-8, **(C)** colony formation assay, and **(D)** Transwell assays were used to assess the proliferation, invasion, and migration of cells. The cell experiment was repeated three times independently and data were all metrological data. All data were shown as mean ± standard deviation. One-way ANOVA was used for data analysis, followed by Tukey’s test. *P < 0.05, ***P < 0.001.

To confirm that SOX2-OT could bind to miR-143-3p competitively to regulate the malignant behaviors of HCC cells, a functional rescue experiment was carried to transfect miR-143-3p inhibitor into HepG2 cells with si-SOX2-OT. Firstly, RT-qPCR found that miR-143-3p was downregulated significantly after the transfection of miR-143-3p inhibitor (*p* < 0.001, **Figure 4A**). CCK-8 and colony formation assays demonstrated that after the transfection of miR-143-3p inhibitor, cell proliferation was enhanced. The downregulation of miR-143-3p reversed the changes of cell proliferation mediated by si-SOX2-OT#2 (all *p* < 0.001, **Figures 4B, C**). Transwell assays indicated that after the transfection of miR-143-3p inhibitor, cell invasion and migration were enhanced, and the decreases of cell migration and invasion mediated by si-SOX2-OT#2 were also reversed (*p* < 0.001, **Figure 4D**). These results showed that SOX2-OT competitively bound to miR-143-3p and promoted the biological behaviors of HCC cells.

### Upregulation of MSI2 Averted the Effect of si-SOX2-OT on HCC

**Figure 5:**
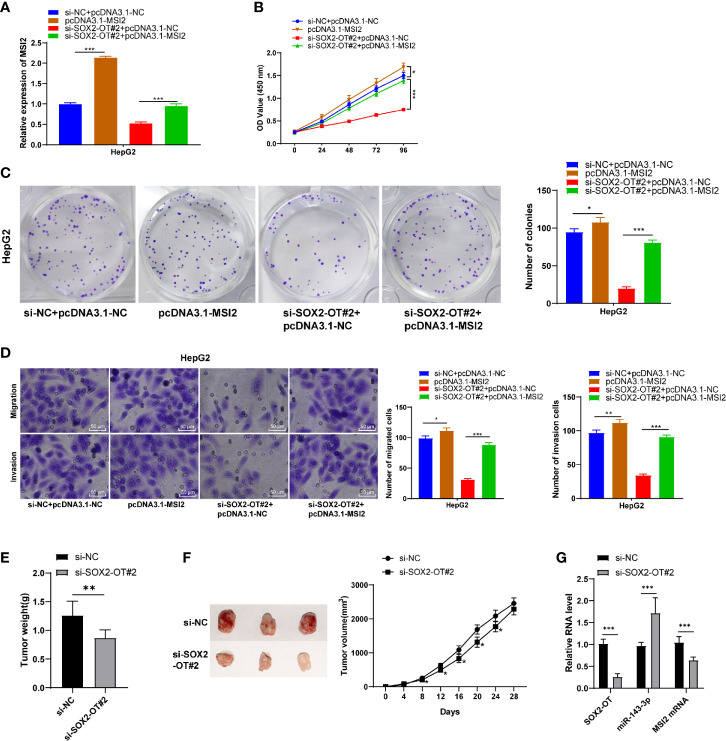
Upregulation of MSI2 could reverse the role of si-SOX2-OT in HCC. pcDNA3.1-MSI2 was delivered into HepG2 cells which were transfected with si-SOX2-OT#2, and the cells with si-NC + pcDNA3.1-NC were used as control. Then, **(A)** RT-qPCR was used to verify the intervention effect of pcDNA3.1-MSI2; **(B)** CCK-8, **(C)** colony formation assay, and **(D)** Transwell assays were utilized to assess proliferation, invasion, and migration of the cell; **(E)** After 4 weeks of subcutaneous tumorigenesis in nude mice, tumor bodies were obtained and tumor weight was measured; **(F)** Changes of tumor volume within 4 weeks after subcutaneous tumorigenesis in nude mice and typical pictures; **(G)** The levels of SOX2-OT, miR-143-3p and MSI2 protein in tumor bodies were detected by RT-qPCR. There were 6 mice in each group. The cell experiment was repeated three times independently and data were all metrological data. All data were shown as mean ± standard deviation. One-way ANOVA was used for data analysis, followed by Tukey’s test. *P < 0.05, **P < 0.01, ***P < 0.001.

To further verify the ceRNA mechanism of the SOX2-OT/miR-143-3p/MSI2 in HCC, a functional rescue experiment was conducted to study the change of HCC cell malignant behaviors after the intervention of MSI2 expression. Firstly, RT-qPCR showed that MSI2 in HCC cells was upregulated significantly after transfection of pcDNA3.1 MSI2 (*p* < 0.001, **Figure 5A**). Overexpression of MSI2 could promote cell proliferation and reverse changes of cell malignant behaviors mediated by si-SOX2-OT#2 (all *p* < 0.001, **Figures 5B, C**). Transwell assays showed that overexpression of MSI2 also enhanced cell invasion and migration, and reversed si-SOX2-OT#2-mediated cell invasion and migration (all *P* < 0.001, **Figure 5D**). *In vivo* experiments showed that the tumor volume and weight in the si-SOX2-OT#2 group were significantly decreased compared with the control group (all *P* < 0.05, **Figures 5E, F**), and there were significant differences in miR-143-3p and MSI2 mRNA levels in tumor tissues (all *P* < 0.001, **Figure 5G**). Briefly, SOX2-OT could bind to miR-143-3p competitively to reduce the targeted inhibition of miR-143-3p on MSI2 and promote MSI2, and finally promote the malignant episodes of HCC cells.

## Discussion

HCC, primarily caused by chronic hepatitis, subsequent cirrhosis, and fibrosis, is the most deadly kind of liver cancer worldwide (30). The treatment methods for patients with advanced HCC are limited at present (5). Evidence has shown that lncRNA SOX2 is upregulated in HCC tissues (31). It is promising to explore effective treatments for HCC with lncRNA SOX2 as a breakthrough point. This study elucidated that lncRNA SOX2-OT could promote HCC cell malignant behaviors *via* the miR-143-3p/MSI2 axis.

It is known that dysregulation of lncRNA SOX2 is related to a multitude of cancers (32). Some recent studies have shown that SOX2-OT upregulation is related to the occurrence and prognosis of cervical cancer, A375-M6 melanoma, and prostate cancer (33–35). However, the SOX2-OT expression in HCC was still unclear. This study indicated that SOX2-OT was upregulated in HCC cells and tissues, indicating that SOX-OT might be related to HCC occurrence. Additionally, we analyzed the relationship between SOX2-OT expression and prognosis of HCC patients using the TCGA visualization website Starbase. The results demonstrated that there was no significant difference in the K-M curve of patients with different SOX2-OT expressions, but the long-term prognosis of patients with low SOX2-OT expression showed a better trend. However, in a previous study, the 5-year overall survival of patients with high expression of lncRNA SOX2-OT was significantly shorter than that of the patients with low lncRNA SOX2-OT expression (36). Patients with higher lncRNA SOX2-OT expression were predicted to have a relatively poor disease-free survival and short overall survival time compared with patients with lower lncRNA SOX2-OT expression (37). Therefore, we cannot deny the potential role of lncRNA SOX2-OT in the prognosis of HCC. Consistently, the aberrantly expressed SOX genes have been found in clinical HCC patients; and the SOX signature genes are found to be related to tumor grade and tumor stage (38). In brief, SOX2-OT might be associated with the occurrence and prognosis of HCC. Then we explored the function of SOX-OT in HCC cell malignant behaviors. RT-qPCR demonstrated that SOX2-OT expression was significantly decreased after transfection of SOX2-OT siRNA. After the downregulation of SOX2-OT, malignant behaviors of HCC cells were inhibited. It has been revealed that SOX2-OT knockdown can inhibit cell proliferation, arrest the cell cycle, facilitate apoptosis, and inhibit the metastasis of nasopharyngeal carcinoma (12). Hence, we concluded that the downregulation of SOX2-OT could prevent malignant biological behaviors of HCC cells.

LncRNA can promote cell malignant behaviors by acting as a ceRNA in HCC cells (39). We then explored the potential ceRNA mechanism of SOX2-OT in HCC. SOX2-OT was mainly distributed in the cytoplasm of Huh7 and HepG2 cells, and the binding relations of SOX2-OT and miR-143-3p, and miR-143-3p and MSI2 were predicted and verified by the RNA pull-down assay and dual-luciferase assay. The miR-143-3p in HepG2 and Huh7 cells with si-SOX2-OT#2 was upregulated clearly and MSI2 was downregulated. A recent study indicates that SOX2-OT acts as a ceRNA to sponge the miR-144-3p (22). The target relationship of miR-143-3p and MSI2 is verified in another study (25). Altogether, SOX2-OT sponged miR-143-3p and promoted MSI2.

Furthermore, the miR-143-3p inhibitor was transfected into HepG2 cells with silencing SOX2-OT in the functional rescue experiments, and then the role of miR-143-3p in HCC cells was explored. miR-143-3p expression was decreased after the transfection with miR-143-3p inhibitor. Downregulation of miR-143-3p reversed the changes of cell malignant behaviors mediated by si-SOX2-OT#2, which further confirmed the conclusion that SOX2-OT sponged miR-143-3p and promoted MSI2. A study shows that upregulation of miR-143-3p inhibits cell malignant behaviors, and is identified to be a potential therapeutic target for HCC (40). This study supported that downregulation of miR-143-3p could avert the inhibitory effect of si-SOX2-OT on HCC cells.

The ceRNA mechanism of SOX2-OT/miR-143-3p/MSI2 in biological behaviors of HCC cells was further verified using rescue experiments. RT-qPCR showed that MSI2 in HCC cells was notably increased after the transfection of pcDNA3.1 MSI2. Overexpression of MSI2 reversed the changes of cell malignant behaviors mediated by si-SOX2-OT#2. A study shows that MSI2 is positively correlated with high CD44v6 expression, which can promote the malignant behaviors of HCC cells (29). Collectively, our results confirmed that SOX2-OT could promote MSI2 by binding to miR-143-3p competitively, and finally promote the malignant behaviors of HCC cells. There are few studies about the ceRNA mechanism of SOX2-OT/miR-143-3p/MSI2 in HCC cells, which suggests the novelty of our study.

In summary, this study supported that lncRNA SOX2-OT promoted proliferation and invasion of HCC cells *via* the ceRNA mechanism involved miR-143-3p/MSI2. This study simply revealed that lncRNA SOX2-OT might participate in the biological progress of HCC cells *via* the ceRNA network, but didn’t reveal whether lncRNA SOX2-OT and miR-173-3p could be biomarkers for early screening of HCC. Further work is required to study the regulatory mechanism of lncRNA SOX2-OT on HCC from the perspective of epigenetics.

## Data Availability Statement

The original contributions presented in the study are included in the article/supplementary material. Further inquiries can be directed to the corresponding author.

## Ethics Statement

The studies involving human participants were reviewed and approved by Affiliated Xinxiang Central Hospital of Xinxiang Medical University. The patients/participants provided their written informed consent to participate in this study.

## Author Contributions

HZ is the guarantor of integrity of the entire study. HZ contributed to the study concepts, study design, the definition of intellectual content, manuscript preparation, and manuscript editing and review. MB contributed to the clinical studies. ML contributed to the experimental studies. XY contributed to the data analysis and data acquisition. LS contributed to the statistical analysis. All authors contributed to the article and approved the submitted version.

## Funding

This study was supported by Xinxiang Major S&T Project, The basic and clinical research of microwave ablation’s effects on cellular immunity and peritumor microenvironment in liver cancer, Grant/Award Number: ZD1402; The innovation funds of health science and technology providing for Henan young and middle-aged doctors. 

## Conflict of Interest

The authors declare that the research was conducted in the absence of any commercial or financial relationships that could be construed as a potential conflict of interest.
